# Supplementary material for: Diagnostic performance and clinical implications of rapid SARS-CoV-2 antigen testing in Mexico using real-world nationwide COVID-19 registry data
Source: PLoS One. 2021 Aug 31;16(8):e0256447. doi: 10.1371/journal.pone.0256447 (PMC8407542; doi:10.1371/journal.pone.0256447)
Supplement: Attachment — Submitted filename: Reviewer PlosOne PONE-D-21-07222 yb.docx [file pone.0256447.s003.docx]

Research article: PONE-D-21-07222

Title: Diagnostic performance and clinical implications of rapid SARS-CoV-2 antigen testing

in Mexico using real-world nationwide COVID-19 registry data

Bello-Chavolla and colleagues have submitted an interesting and well written article presenting a retrospective study assessing the Diagnostic performance and clinical implications of rapid SARS-CoV-2 antigen testing that was performed in Mexico. Using more than 18,000 patients tests by qRT-PCR and Rapid Antigen tests, they conclude that the Rapid Ag-T could be used as an alternative to qRT-PCR for large scale SARS-CoV-2 testing in Mexico. However they highlight that the interpretation of Rapid Ag-T results should be done with caution in order to minimize the risk associated with what they consider false results based on the qRT-PCR use as reference.

There are however some points to address before being considered for publication in PlosOne

1. The authors should add the actual “optimal” performance of the Rapid Ag-T between 7-10 days after symptoms in the abstract
2. In the line 192 there are mentioned positive and negative results and I was wondering if there were no indeterminate results and what was the process to handle the indeterminate
3. In line 204, the authors mentioned that 18,446 cases had both rapid test and qRT-PCR but it would be great to mention if the samples were collected at the same time and if not what was the delay between the sample collection and the test knowing that it might affect the comparison
4. In line 205 the authors mentioned “the cases” but need to explain who they actually talk about.
5. In line 208 it would be great to the percentage of each group for ease of reading
6. Do the author have more information about the correlation between the rapid test and the Ct Value of the PCR. This is an important information in how to consider the performance of the rapid tests vs PCR
7. In line 274 the author should consider the published algorithm <https://www.thelancet.com/pdfs/journals/laninf/PIIS1473-3099(21)00132-8.pdf> . The authors should compare their results to field evaluation of rapid tests in asymptomatic done in Cameroon and the suggested algorithm to see what could be proposed to Mexico
